# Mechanism of Microbicidal Action of E-101 Solution, a Myeloperoxidase-Mediated Antimicrobial, and Its Oxidative Products

**DOI:** 10.1128/IAI.00261-19

**Published:** 2019-06-20

**Authors:** Gerald A. Denys, Neil C. Devoe, Polyxeni Gudis, Meghan May, Robert C. Allen, Jackson T. Stephens

**Affiliations:** a Indiana University School of Medicine, Indianapolis, Indiana, USA; b University of New England College and Osteopathic Medicine, Biddeford, Maine, USA; c Creighton University School of Medicine, Omaha, Nebraska, USA; d Exoxemis, Inc., Little Rock, Arkansas, USA; University of Illinois at Chicago

**Keywords:** E-101 solution, antimicrobial agent, hydrogen peroxide, hypochlorous acid, myeloperoxidase, oxidants, singlet oxygen

## Abstract

E-101 solution is a first-in-class myeloperoxidase-mediated antimicrobial developed for topical application. It is composed of porcine myeloperoxidase (pMPO), glucose oxidase (GO), glucose, sodium chloride, and specific amino acids in an aqueous solution. Once activated, the reactive species hydrogen peroxide (H_2_O_2_), hypochlorous acid, and singlet oxygen are generated. We evaluated the treatment effects of E-101 solution and its oxidative products on ultrastructure changes and microbicidal activity against methicillin-resistant Staphylococcus aureus (MRSA) and Escherichia coli. Time-kill and transmission electron microscopy studies were also performed using formulations with pMPO or GO omitted. The glutathione membrane protection assay was used to study the neutralization of reactive oxygen species. The potency of E-101 solution was also measured in the presence of serum and whole blood by MIC and minimal bactericidal concentration (MBC) determinations. E-101 solution demonstrated rapid bactericidal activity and ultracellular changes in MRSA and E. coli cells. When pMPO was omitted, high levels of H_2_O_2_ generated from GO and glucose demonstrated slow microbicidal activity with minimal cellular damage. When GO was omitted from the formulation, no antimicrobial activity or cellular damage was observed. Protection from exposure to E-101 solution reactive oxygen species in the glutathione protection assay was competitive and temporary. E-101 solution maintained its antimicrobial activity in the presence of inhibitory substances, such as serum and whole blood. E-101 solution is a potent myeloperoxidase enzyme system with multiple oxidative mechanisms of action. Our findings suggest that the primary site where E-101 solution exerts microbicidal action is the cell membrane, by inactivation of essential cell membrane components.

## Abstract (precis)

E-101 solution is a first-in-class myeloperoxidase-mediated antimicrobial developed for topical application. It is composed of porcine myeloperoxidase (pMPO), glucose oxidase (GO), glucose, sodium chloride, and specific amino acids in an aqueous solution.

## INTRODUCTION

**FIG 1:**
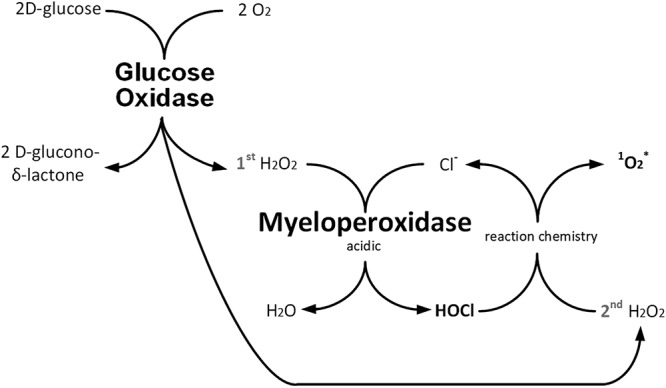
Schematic depicting the enzyme-linked oxidative action of E-101 solution. E-101 solution is composed of GO and pMPO in an aqueous vehicle and activated by the addition of glucose. Diradical molecular oxygen (O_2_) is reduced to nonradical diamagnetic singlet multiplicity H_2_O_2_, myeloperoxidase catalyzes the oxidation of nonradical diamagnetic singlet multiplicity Cl^−^ to nonradical singlet multiplicity HOCl, and the H_2_O_2_-HOCl reaction yields electronically excited diamagnetic singlet multiplicity O_2_. H_2_O_2,_ hydrogen peroxide; Cl^−^ chloride; HOCl, hyphochlorous acid; ^1^O_2_, singlet molecular oxygen.

E-101 solution is a first-in-class topical myeloperoxidase (MPO)-mediated formulation developed as an antimicrobial open-wound wash solution. E-101 solution is not intended to be given systemically. It is composed of two enzymes, glucose oxidase (GO) and porcine myeloperoxidase (pMPO), in an aqueous vehicle. Upon topical application of E-101 solution containing glucose, hydrogen peroxide (H_2_O_2_) is produced *in situ* by GO, which drives pMPO-dependent oxidation of chloride to hypochlorous acid (HOCl). Once generated, HOCl (or its conjugate base, OCl^−^ [pK_a_ = 7.5]) participates in a diffusion-controlled reaction with a second H_2_O_2_ molecule to yield singlet molecular oxygen (^1^O_2_), a metastable electronically excited reactant (Fig. 1). Singlet oxygen is a potent electrophilic oxygenating agent capable of reacting with a broad spectrum of electron-rich compounds. Singlet oxygen has a microsecond lifetime that restricts combustive oxygenation to the proximity of its generation. The bactericidal activity of the MPO antimicrobial system is enhanced by the selective binding of MPO to the surfaces of target microorganisms (1–3). Selective MPO binding results in selective microbicidal action with minimal bystander damage. *In vivo* and *in vitro* studies have shown that E-101 solution exerts potent and broad-spectrum microbicidal action against Gram-positive and Gram-negative bacteria, including multidrug-resistant pathogens (4–7).

The microbicidal combustive action of E-101 solution against target microorganisms is thought to be directed to a variety of molecular and enzymatic sites that are essential for metabolism or for the integrity of the microorganism (8). As part of new product development, this study was undertaken to better understand the potential mechanism(s) of action of E-101 solution and inhibition of activity. The goals of this study were (i) to determine the time-kill effects of E-101 solution and its oxidative products on the ultrastructure of Gram-positive and Gram-negative bacteria, (ii) to determine the oxidative effect of E-101 solution on cellular damage using the glutathione membrane protection assay, and (iii) to compare the rate of killing of E-101 solution to that of sodium oxychlorosene in the absence and in the presence of serum and whole blood.

(This work was presented in part at the 21st European Congress of Clinical Microbiology and Infectious Diseases and the 27th International Congress of Chemotherapy, Milan, Italy, 2011 [9], and the 115th General Meeting of the American Society for Microbiology, New Orleans, LA, 2015 [10].)

## RESULTS

### Time-kill effect of E-101 solution and oxidative intermediates.

The bactericidal activity of E-101 solution was dependent on all the components of the antimicrobial system (pMPO, H_2_O_2_, and halide). Time-kill curves of methicillin-resistant Staphylococcus aureus (MRSA) demonstrated rapid bactericidal activity of complete E-101 solution at 100, 416, and 833 μg pMPO/ml (Table 1). At the early 5-min measurement, the rate of MRSA killing was inversely proportional to the E-101 solution concentration, but the 30- and 60-min measurements showed extensive 6-log-unit killing. For Escherichia coli, the 5-min and 30-min measurements of killing were directly proportional to the E-101 solution concentration, and at 60 min, all the concentrations showed extensive 6-log-unit killing. The differences in early kill rates with respect to S. aureus and E. coli may reflect inhibition of MPO haloperoxidase activity by higher concentrations of H_2_O_2_ generated by GO at the higher E-101 concentrations tested. S. aureus has high catalase expression, whereas E. coli has relatively low catalase expression. MPO is inhibited by concentrations of H_2_O_2_, especially if the ratio of H_2_O_2_ to chloride is high (11, 12).

**TABLE 1:** Comparative time-kill of MRSA and E. coli exposed to complete E-101 solution, enzyme solution containing GO only, and enzyme solution containing pMPO only

Enzyme solution	Enzyme concn (μg/ml)^*a*^		Time (min)	Log kill	
	pMPO	GO		MRSA	E. coli
Complete E-101	100	20	5	1.5 × 10^4^	1.2 × 10^4^
	416	83	5	7.8 × 10^1^	1.2 × 10^4^
	832	167	5	1.3 × 10^1^	2.3 × 10^5^
	100	20	30	1.5 × 10^6^	1.7 × 10^4^
	416	83	30	6.7 × 10^5^	1.2 × 10^6^
	832	167	30	1.3 × 10^6^	2.3 × 10^6^
	100	20	60	1.5 × 10^6^	1.2 × 10^6^
	416	83	60	6.7 × 10^5^	1.2 × 10^6^
	832	167	60	1.3 × 10^6^	2.3 × 10^6^
GO	0	20	5	0	<1
	0	83	5	<1	<1
	0	167	5	<1	<1
	0	20	30	0	<1
	0	83	30	<1	<1
	0	167	30	<1	2.7 × 10^1^
	0	20	60	<1	1.5 × 10^3^
	0	83	60	<1	2.0 × 10^4^
	0	167	60	<1	2.3 × 10^6^
pMPO	100	0	5	0	0
	416	0	5	0	0
	832	0	5	0	0
	100	0	30	0	0
	416	0	30	0	0
	832	0	30	0	0
	100	0	60	0	0
	416	0	60	0	0
	832	0	60	0	0

a The concentrations of pMPO tested were proportional to the GO concentrations.

In the absence of pMPO, H_2_O_2_ generated from GO and glucose showed no activity against MRSA at 20 μg GO/ml but some antimicrobial activity at 83 and 167 μg GO/ml, especially against E. coli. The bactericidal activity of H_2_O_2_ was observed at 167 μg GO/ml, but at a lower rate (60 min). The pMPO-H_2_O_2_ microbicidal action was several orders of magnitude more rapid and more potent than that of H_2_O_2_ alone. The catalase content of S. aureus has been reported to be a virulence factor (13). S. aureus catalase competitively destroys H_2_O_2_, lowering its concentration without adversely affecting the rapid activity of E-101 solution. Sufficient H_2_O_2_ is present to drive the formation of both HOCl and ^1^O_2_, which are thought to be the microbicidal agents in the system. However, in the absence of H_2_O_2_ generated by GO glucose-catalyzed reduction of oxygen, pMPO is unable to exert any antimicrobial activity against catalase-positive MRSA.

The time-kill curves of E. coli ATCC 25922 (Table 1) demonstrated similar rapid bactericidal activities of complete E-101 solution (pMPO plus GO) at all three pMPO concentrations. High levels of H_2_O_2_ generated from GO and glucose in the absence of MPO showed bactericidal activity, but at a rate orders of magnitude lower than the complete MPO-containing E-101 solution. Unlike S. aureus, E. coli, a weak catalase producer, showed susceptibility to H_2_O_2_ generation over time and at high concentrations. Formulation without GO added demonstrated no antimicrobial activity at all pMPO concentrations tested.

### Ultrastructure effect of E-101 solution and oxidative intermediates.

**FIG 2:**
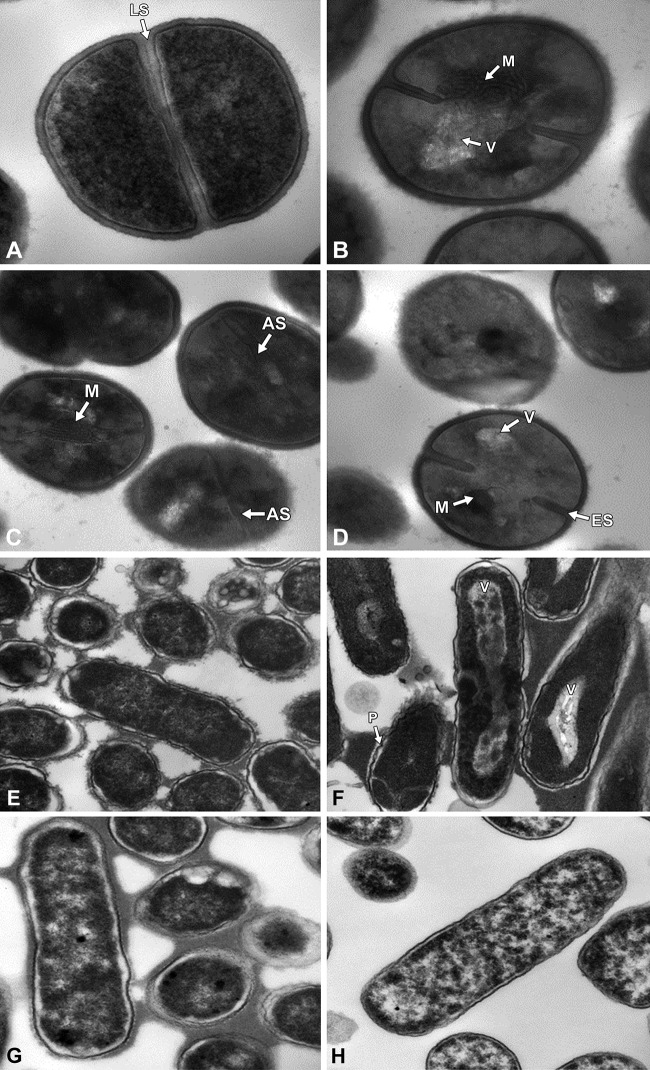
(A to D) Morphological changes of MRSA cells harvested from exponential growth phase exposed to complete E-101 solution. (A) Untreated control cells of MRSA showed uniform septa (LS). Magnification, ×68,000. (B) MRSA treated with complete E-101 solution at 120 min. Magnification, ×68,000. (C and D) MRSA cells treated with complete E-101 solution at 120 (C) and 60 (D) min. Magnification, ×49,000. The treated cells showed early septa (ES), cytoplasmic vacuoles (V), cytoplasmic mesosomes (M), and aberrant septa (AS). (E to H) Morphological changes of E. coli cells harvested from exponential growth phase exposed to complete E-101 solution, formulation containing GO only, and formulation containing pMPO only. (E) E. coli cells treated with complete E-101 solution demonstrated no morphological changes at initiation of treatment. Magnification, ×23,000. (F) Treatment of E. coli cells with complete E-101 solution at 120 min caused large elongated cytoplasmic vacuoles (V) and pleated cells (P). Magnification, ×23,000. (G and H) E. coli cells treated with formulation containing pMPO only (no GO) (G) or formulation containing GO only (no pMPO; H_2_O_2_ generated) (H) at 120 min demonstrated generalized vacuolization after prolonged exposure but no abnormal cytoplasmic membrane or cell wall changes. Magnification, ×30,000.

Exposure of MRSA to complete E-101 antimicrobial solution at concentrations of 416 and 833 μg pMPO/ml and 83 and 167 μg GO/ml for up to 120 min induced morphological changes (Fig. 2). Both time-kill and transmission electron microscopy (TEM) studies revealed that complete E-101 solution generates oxidative products that damage cells in a time- and concentration-dependent manner when pMPO, halide, and a source of hydrogen peroxide are present. At 30 min, the majority of cells appeared ultrastructurally normal, i.e., the cell wall, cell membrane, and major constituents of the cell cytoplasm appeared similar to those of the control, but the microbes were killed, as measured by CFU activity. However, post-kill ultrastructural changes were noted after 1 and 2 h, mainly at the cell membrane level. At 60 and 120 min, mesosome-like structures and septal defects in MRSA appeared. At 120 min, septal defects were more pronounced, displaying thickened septa. Changes in the cytoplasmic membrane, mesosome-like structures, did not occur in untreated S. aureus cells. Increased vacuolation of the cytoplasm was also seen at 60 and 120 min. Similar morphological changes were observed in antibiotic-treated S. aureus (14, 15). No apparent ultrastructure changes were observed in cultures treated with enzyme formulations when GO or pMPO was omitted (not shown).

Exposure of E. coli to complete E-101 also induced time- and concentration-dependent morphological changes in septal formation and cytoplasm at 60 and 120 min (Fig. 2). E-101 solution induced cellular changes in 50% to 75% of cells that included septal deformation, elongation of cytoplasmic vacuoles, and pleated cell walls in E. coli. These post-kill findings are consistent with the activity of E-101 solution and the generation of reactive species, H_2_O_2,_ HOCl, and ^1^O_2_. Cultures treated with partial formulations containing the enzyme GO or pMPO alone demonstrated increased vacuolization. No abnormal cytoplasmic membrane or cell wall changes were observed when pMPO or GO was omitted from the formulation. The lack of obvious ultrastructure changes but extensive 6-log-unit killing at 30 min of treatment with E-101 solution suggest that killing involves the combustive denaturation of key enzymatic components and/or destruction of membrane integrity that precedes ultrastructural damage.

### Oxidative destruction of glutathione by E-101 solution.

**FIG 3:**
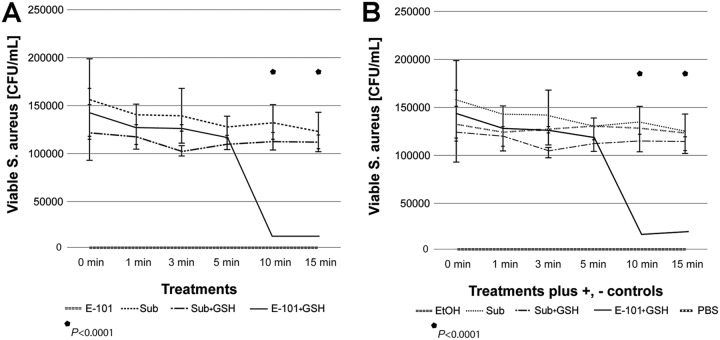
Glutathione membrane protection assay. (A) Glutathione treatment. (B) Glutathione treatment plus controls. Exposure of S. aureus to E-101 solution in the presence of 50 mM glutathione depleted the time required to achieve oxidative destruction of glutathione. A significant (*P* < 0.0001) reduction in viability was seen only after 10 min of exposure compared to controls. E-101, E-101 solution; Sub, E-101 substrate alone; Sub + GSH, E-101 substrate plus glutathione; E-101 + GSH, E-101 solution plus glutathione. Positive control, EtOH; negative control, PBS. Error bars represent standard deviations.

The presence of 50 mM glutathione delayed but did not prevent E-101 solution killing of S. aureus. A significant (*P* < 0.0001) reduction in viability was seen only after 10 min of exposure (Fig. 3).

### Effects of inhibitors on the myeloperoxidase antimicrobial system.

**FIG 4:**
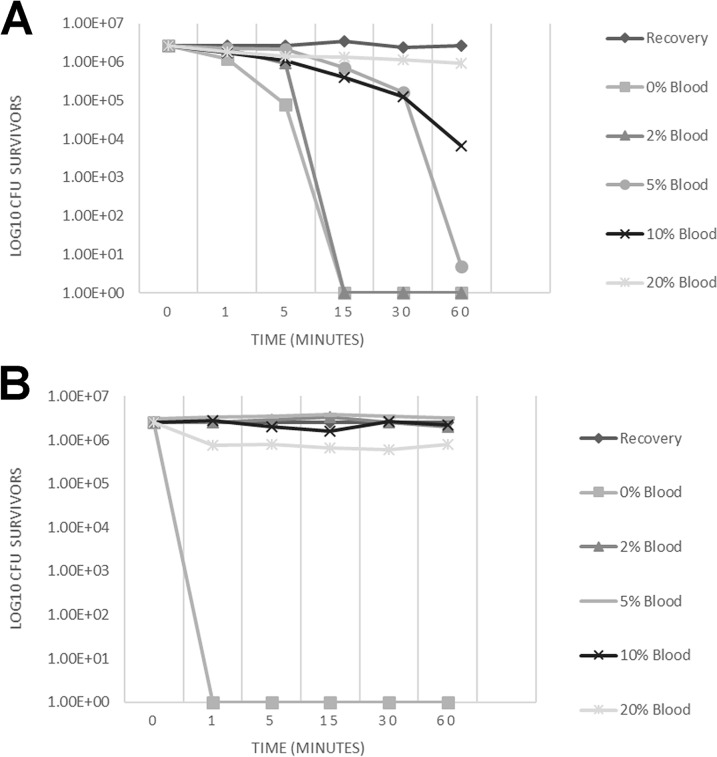
(A) Time-kill results for MRSA treated with complete E-101 at 0.83 mg pMPO/ml showed bactericidal activity (≥5 log_10_ reduction) in the presence of 2 and 5% whole human blood, with continued activity in the presence of 10% blood. No activity was observed in the presence of 20% whole human blood. (B) Time-kill results for MRSA treated with sodium oxychlorosene at 4 mg/ml showed rapid killing within 1 min in the absence of whole human blood but no activity in the presence of ≥2% whole human blood.

The *in vitro* activity of the complete E-101 solution was compared to that of sodium oxychlorosene (Clorpactin WCS-90), a buffered hypochlorous acid formulation, in the presence of serum and whole human blood. Table 2 summarizes the MICs and minimal bactericidal concentrations (MBCs) of both agents against MRSA and E. coli. Both E-101 solution and sodium oxychlorosene were highly active in the absence of serum or blood. However, in the presence of serum or blood, E-101 solution maintained a high level of activity, whereas sodium oxychlorosene activity was completely inhibited. The active component of sodium oxychlorosene is HOCl. Based on time-kill kinetic data in the absence of serum or blood, the rate of killing was slightly higher for sodium oxychlorosene (1 versus 15 min); however, E-101 solution demonstrated sustained and more potent killing than sodium oxychlorosene against S. aureus in the presence of 2, 5, and 10% blood (Fig. 4). This is consistent with the selective binding and microbicidal action of MPO (1). The reaction of HOCl with additional H_2_O_2_ yields ^1^O_2_ with a microsecond lifetime that restricts killing to the proximity of MPO binding. The potent oxidative antibacterial activity of E-101 solution was less susceptible than that of sodium oxychlorosene to the inhibitory effect of blood containing catalase and other competitive substances.

**TABLE 2:** Comparative *in vitro* activities of complete E-101 solution and sodium oxychlorosene in the presence of horse serum and human whole blood

Organism	% serum	% blood	E-101^*a*^		Sodium oxychlorosene^*b*^	
			MIC (mg/ml)	MBC (mg/ml)	MIC (mg/ml)	MBC (mg/ml)
MRSA	0		0.000008	0.000008	0.03	0.03
	10		0.000008	0.000008	>2	>2
	20		0.000008	0.000008	>2	>2
		0		<0.00012		0.015
		2		0.0080		>2
		5		0.0320		>2
		10		0.0320		>2
		20		0.0640		>2
		0				
E. coli	0		0.00025	0.00025	0.06	0.06
	10		0.00025	0.00025	>2	>2
	20		0.00050	0.00050	>2	>2
		0		0.0005		0.004
		2		0.0320		>2
		5		0.0320		>2
		10		0.0640		>2
		20		0.1280		>2

a E-101 range for MIC, 0.008 to 0.000008 mg pMPO/ml; E-101 range for MBC, 0.128 to 0.00012 mg pMPO/ml.

b Sodium oxychlorosene range for MIC, 2.0 to 0.002 mg/ml; sodium oxychlorosene range for MBC, 2.0 to 0.002 mg/ml.

## DISCUSSION

MPO is a heme-containing haloperoxidase present in high concentrations in neutrophilic granulocytes. MPO is one of the key components of the oxygen-dependent antimicrobial system of the neutrophil phagosome (16). The importance of MPO in killing of phagocytosed pathogens has been well documented (17, 18). The ability to efficiently kill phagocytosed microbes requires the activities of membrane-bound NADP oxidase (NADPH oxidase) and MPO within the phagolysosome. NADPH oxidase is a flavocytochrome enzyme capable of univalent reduction of molecular oxygen (O_2_), yielding the acid hydroperoxyl radical (HO_2_) and its conjugate base, the superoxide anion (O_2_^−^). In the phagolysosomal system HO_2_-O_2_^−^, disproportionation yields the H_2_O_2_ required for myeloperoxidase oxidation of chloride, yielding HOCl. HOCl chemically reacts with a second H_2_O_2_, yielding ^1^O_2_. Both HOCl and ^1^O_2_ are potent microbicidal agents (1, 19–22). The highly electrophilic reactivity of ^1^O_2_ enables it to oxidize regions of high electron density in target biological molecules, resulting in destruction of membrane integrity and/or the oxidative deactivation of the enzymes required for metabolic function.

*In vitro* studies have confirmed that the combination of MPO, its substrate H_2_O_2_, and a halide forms a potent antimicrobial system (23). The MPO-H_2_O_2_-halide system is capable of generating a wide range of oxidant species, including ^1^O_2_, HOCl, and chloramines (16). All of these products are reactive, but only ^1^O_2_ is electronically excited, with a half-life of about a microsecond. OCl^−^ salts and chloramines can be stored for years. These highly reactive compounds can oxidize susceptible reactive groups in biochemical substrates, including thiols, thioesters, heme groups, and unsaturated fatty acids (24). Such oxidative modifications alter protein and lipid membrane activities and consequently affect microbial and cellular functions, including membrane structure and metabolic activity.

E-101 solution is a formulated cell-free oxidant-generating enzyme system. E-101 solution consists of two enzymes, i.e., pMPO and GO, with glucose as a substrate plus sodium chloride and proprietary amino acids. The oxidant-generating enzyme system is activated upon mixing of the components. Although E-101 solution mimics the intrinsic functions of the phagolysosome, there is a significant difference between the systems. The E-101 solution substitutes GO for the NADPH oxidase of the phagolysosomal system, and as such, no acid hydroperoxyl radical (HO_2_) or superoxide anion (O_2_^−^) is generated. GO, a bimolecular flavoprotein enzyme, catalyzes the two equivalent reductions of O_2_, directly producing the H_2_O_2_ required for pMPO oxidation of chloride. The present study demonstrates that the microbicidal activity of E-101 solution is dependent on each component of the functional system. In the absence of pMPO, antimicrobial activity is greatly decreased. The absence of GO also greatly decreases antimicrobial activity.

The microbicidal mechanism of action by E-101 solution involves binding of pMPO to the surfaces of target microorganisms, where HOCl and ^1^O_2_ focus direct oxidative damage (1, 2, 4, 25, 26). In the presence of H_2_O_2_, generated *in situ* by GO from glucose and oxygen, the microorganism-bound pMPO catalyzes the oxidation of chloride ion to HOCl. Hypochlorite reacts with an additional H_2_O_2_, yielding ^1^O_2_ at or near the surface of the target organism.

In the glutathione membrane protection assay, the initial protective effect suggests that glutathione, a sulfhydryl-reducing agent, neutralizes the oxidative and deoxygenating agents generated from E-101 solution. The loss of inhibition after a 10-minute lag period suggests that once oxidative depletion of 50 mM glutathione is complete, oxidation of the target bacteria follows, resulting in complete killing. This indicates that the protection provided by glutathione is competitive and temporary.

Ultrastructural evidence follows the microbicidal action. The time-kill results demonstrated that E-101 solution kills within minutes of direct exposure and that this microbicidal action is rapid and precedes any observable disruption of the microbial plasma membrane, suggesting oxidative inhibition of vital membrane enzyme systems. E-101 solution induced subtle but clearly visible changes in bacterial cells at longer incubation times. Active pMPO was essential to demonstrating cell damage, since no damage was seen when pMPO was omitted from the formulation. Similar studies support these finding in damaging *Candida* hyphae and pseudohyphae. Oxidative intermediates appeared to damage hyphae only if MPO, halide, and a source of H_2_O_2_ were present or if ^1^O_2_ was produced (27).

Consistent with the selective binding and focused combustive oxygenation mechanism of action, E-101 solution was less susceptible to the inhibitory effect of blood containing catalase and other substances that competitively react with available ^1^O_2_ and HOCl (12). Microorganisms such as E. coli and S. aureus, with strong MPO binding capability, are targeted for MPO-generated HOCl and ^1^O_2_. In contrast, sodium oxychlorosene, with the active component HOCl, was rapidly bactericidal but easily inactivated by the presence of serum or blood. Previous studies have demonstrated that the microbicidal activity of MPO-H_2_O_2_ is several orders of magnitude more potent than that of H_2_O_2_ alone and more resistant to erythrocyte inhibition than the activity of either H_2_O_2_ or HOCl (1, 2).

In summary, our data demonstrate that the bactericidal action of E-101 solution is attributable to the MPO-derived oxidants generated from the pMPO-H_2_O_2_-Cl antimicrobial system. E-101 solution reacts with the bacterial membrane components that are essential for viability and cell division. This enzyme system may play an important role in antimicrobial therapy.

## MATERIALS AND METHODS

### Reagents and enzymes.

Stock solutions of complete E-101 solution containing pMPO and GO, enzyme solution containing GO, enzyme solution containing pMPO, and substrate solution containing glucose were prepared at Exoxemis, Inc. (Omaha, NE). A range of concentrations of enzyme solutions were prepared just prior to use. The complete E-101 solution contains pMPO, GO derived from Aspergillus niger, and proprietary amino acids in an aqueous formulation vehicle consisting of 150 mM sodium chloride and 0.02% (wt/vol) polysorbate 80, pH 6.5, 20 mM sodium, and phosphate buffer. Enzyme solutions containing the individual component GO or pMPO alone were prepared in the same aqueous formulation vehicle. The stock concentrations of pMPO and GO were 2.5 mg/ml and 0.5 mg/ml, respectively. The substrate solution contained 300 mM glucose in the same aqueous formulation as the enzyme solution. The enzyme and substrate solutions were packaged in two separate vials and mixed together to activate the system. The activated formulations were held at room temperature for 15 to 20 min for oxidant generation before adding target bacteria.

Sodium oxychlorosene was obtained from Guardian Laboratories (Hauppauge, NY) and prepared according to the manufacturer’s directions. Donor herd horse serum was obtained from Sigma-Aldrich, Inc. (Milwaukee, WI). Volunteer donor whole blood was obtained from the Blood Bank Department at Indiana University Health Pathology Laboratory (Indianapolis, IN).

### Time-kill assay.

The microbicidal effects of complete E-101 solution and enzyme solutions against MRSA (ATCC 43300) and E. coli (ATCC 25922) were determined by a modified CLSI time-kill assay (28). Several colonies (total, 4 to 6), grown on Trypticase soy agar (TSA) with 5% sheep blood overnight, were suspended in 3 ml of 0.45% sodium chloride, and the suspensions were adjusted to 1.0 McFarland standard. The suspension was then diluted 1:10 in prewarmed Trypticase soy broth (TSB) and incubated for 2 to 4 h at 35°C. When the culture reached its logarithmic growth phase and the turbidity approximated 1.0 McFarland standard, the suspension was diluted 1:5 in normal saline (final concentration, ∼6.0 × 10^7^ CFU/ml). This suspension constituted the inoculum for the time-kill assay. The *in vitro* assays were conducted in glass tubes (20 by 125 mm). The test kill reaction tubes were prepared to contain 4.5 to 5 ml of the appropriate inoculum in saline, substrate, and enzymes (complete E-101 or GO or MPO alone). Complete E-101 solution was mixed 15 min prior to its use. The final concentrations of pMPO in complete E-101 solution and pMPO solution alone were 100, 416, and 833 μg/ml (36, 150, and 300 guaiacol units [GU]/ml). The final concentrations of GO in complete E-101 solution and GO alone were 20, 83, and 167 μg/ml. An identical reaction tube containing saline plus inoculum and substrate solution, but no enzymes, constituted the culture growth control. The final bacterial-cell concentration was ∼10^6^ CFU/ml. The reaction tubes were incubated at 35°C in ambient air, and samples were removed for viable counts at 0, 5, 15, 30, 60, 90, and 120 min. Serial samples were obtained for quantification. A 100-μl sample was immediately removed from each tube at each time point and applied to TSA with 5% sheep blood. Serial 10-fold dilutions were also immediately prepared in sterile saline, and a 100-μl volume of each dilution was applied to duplicate plates containing TSA with 5% sheep blood and spread over the surface with a sterile inoculating loop. The plates at time zero functioned as purity plates. Following overnight incubation at 35°C, colonies were manually counted and viable counts were calculated. The data are presented as log_10_ reductions in CFU per milliliter at designated time points compared to the original number of CFU per milliliter at the start of testing. Bactericidal activity was defined as a 99.9% or a 3-log_10_ CFU/ml reduction in the colony count from the initial inoculum.

### Transmission electron microscopy.

Several colonies (total, 4 to 6) of MRSA (ATCC 43300) and E. coli (ATCC 25922), grown on TSA with 5% sheep blood overnight, were suspended in 3 ml of 0.45% sodium chloride, and the suspensions were adjusted to 1.0 McFarland standard. The cell suspension was then diluted 1:10 in prewarmed TSB and incubated for ∼2 to 4 h at 35°C to logarithmic phase growth. The cell suspension was centrifuged (3,600 rpm for 6 min), washed with normal saline, and resuspended in normal saline to 3 McFarland standard (∼10^9^ CFU/ml). Control cells consisted of equivalent volumes of organism suspension and normal saline with no test article added. Equivalent volumes of organism suspension (e.g., 3 ml) and enzyme formulation (e.g., 3 ml) were combined at the desired concentration (e.g., 100, 416, or 833 μg/ml [36, 150, and 300 GU/ml]). The treated cell suspensions were then incubated at 35°C and sampled at 0, 30, 60, and 120 min. Cells were harvested by centrifugation (10,000 rpm for 10 min) and fixed in 3.0% (vol/vol) glutaraldehyde.

Pellets were fixed for 24 h (minimum) in 3% glutaraldehyde buffered with 0.15 M Na cacodylate, pH 7.2. Following 3 rinses in cacodylate buffer, the pellets were postfixed in 1% osmium tetroxide (aqueous [aq]) for 4 h at room temperature. The pellets were rinsed in buffer and dehydrated through a graded series of ethanol, rinsed twice in propylene oxide, and left overnight in a 1/1 mixture of propylene oxide and Spurrs/Polybed epoxy resin (Electron Microscopy Sciences, Hatfield, PA). The pellets were infiltrated with full-strength resin for 8 h prior to embedding them in beam capsules with fresh resin. The specimens were polymerized overnight in a 70°C oven. Thin sections of the pellets were placed on 200 mesh cooper grids and stained with lead citrate and 3% uranyl acetate (aq). The sections were examined with an FEI Tecnai G^2^ Spirit transmission electron microscope operated at 80 kV. Images were captured with an AMT XR 60 digital camera.

### Glutathione membrane protection assay.

Complete E-101 solution was mixed 15 min prior to its use. One milliliter of E-101 active solution was added to 2 ml substrate solution and left to acclimate to room temperature according to the manufacturer’s instructions. An inoculum of S. aureus strain ATCC BAA 1717 was cultured for 4 h to generate actively replicating bacterial cells within a standard incubator; 50 μl of the culture was added to a 3-ml tube for each test condition. Time-kill assays were performed by exposing bacteria for 0, 1, 3, 5, 10, and 15 min to each of the following conditions: E-101 treatment, E-101 substrate alone, E-101 plus glutathione buffer (50 mM glutathione, 75 mM sodium chloride, 1 mM magnesium chloride, 0.1 mM calcium chloride, 80 mM sodium hydroxide, and 10% fetal bovine serum [FBS], pH 8.6) (29), and E-101 substrate plus glutathione buffer. Treatment with 80% ethyl alcohol (EtOH) and phosphate-buffered saline (PBS) served as positive and negative controls, respectively.

Following exposure of S. aureus to each condition for the designated time, the reaction was quenched with the addition of 50 μl of catalase. Bacteria surviving after treatment were enumerated by 10-fold serial dilutions in brain heart infusion (BHI) broth, followed by inoculation of BHI agar. The plates were incubated for 24 h, after which viable colonies were counted to assess the survival rate. Bacteria are reported as CFU per milliliter. The study was performed in triplicate for statistical analysis by analysis of variance (ANOVA).

### MIC determination.

Comparative MIC activities against MRSA (ATCC 43300) and E. coli (ATCC 25922) were determined using a modified broth microdilution method based on CLSI M7 guidelines (5, 30). Modifications included diluting enzyme solutions containing pMPO in double-strength cation-adjusted Mueller-Hinton broth (CAMHB). Next, a standardized bacterial suspension was prepared in double-strength glucose substrate solution containing serum or blood and mixed with serial enzyme dilutions in CAMHB to achieve a final concentration of 5 × 10^5^ CFU/ml. Similarly, sodium oxychlorosene was diluted in double-strength CAMHB and mixed with inoculum containing serum or whole blood. The microdilution panels were incubated in ambient air at 35°C for 18 to 24 h. MICs were determined by observing the lowest concentration of antimicrobial agent that inhibited growth of the organisms. Organisms were tested in the presence of increasing concentrations of horse serum (0, 10, and 20%) and human whole blood (0, 2, 5, 10, and 20%). The test range for complete E-101 solution was 0.128 to 0.00012 mg pMPO/ml, and the test range for sodium oxychlorosene was 2 to 0.002 mg/ml.

### MBC determination.

Comparative MBC activities against MRSA (ATCC 43300) and E. coli (ATCC 25922) were determined in the presence of horse serum and whole blood. A 10-μl sample from the last well of the MIC panel with visible growth and each clear well was plated onto TSA with 5% sheep blood. After incubation in ambient air at 35°C for 18 to 24 h, the plates were examined for growth and colony counts. MBCs were defined as a 99.9% or a 3-log_10_ CFU/ml reduction in the colony count from the initial inoculum.

### Comparative time-kill assay in the presence of serum and whole blood.

The time-kill kinetic studies were conducted against MRSA (ATCC 43300) as described by Tote et al. (31). Reaction tubes were prepared to contain logarithmic-phase growth (10^6^ CFU), the antimicrobial agent, and blood. The final concentrations of activated complete E-101 solution (15-min mix time) and sodium oxychlorosene were 0.83 mg pMPO/ml and 4 mg/ml, respectively. At the desired contact times (1, 5, 15, 30, and 60 min), the test mixture was added to neutralizer solution. After a 5-min incubation, the neutralized mixture was sampled for quantitative culture and incubated at 35°C for 24 h. The log_10_ CFU at each time point was determined and compared to growth controls. Due to in-test dilution, both antimicrobial agents were tested at 80% of the final doses.

### Statistical analysis.

The glutathione membrane protection assay test was performed in triplicate for statistical analysis by ANOVA at each time point using GraphPad Prism version 5.04.
